# Crystal structure, Hirshfeld surface analysis, calculations of inter­molecular inter­action energies and energy frameworks and the DFT-optimized mol­ecular structure of 1-[(1-butyl-1*H*-1,2,3-triazol-4-yl)meth­yl]-3-(prop-1-en-2-yl)-1*H*-benzimidazol-2-one

**DOI:** 10.1107/S2056989024004043

**Published:** 2024-05-14

**Authors:** Zakaria El Atrassi, Mustapha Zouhair, Olivier Blacque, Tuncer Hökelek, Amal Haoudi, Ahmed Mazzah, Hassan Cherkaoui, Nada Kheira Sebbar

**Affiliations:** a Laboratory of Heterocyclic Organic Chemistry, Medicines Science Research Center, Pharmacochemistry Competence Center, Mohammed V University in Rabat, Faculté des Sciences, Av. Ibn Battouta, BP 1014, Rabat, Morocco; b University of Zurich, Department of Chemistry, Winterthurerstrasse 190, CH-8057 Zurich, Switzerland; c Department of Physics, Hacettepe University, 06800 Beytepe, Ankara, Türkiye; d Laboratory of Applied Organic Chemistry, Sidi Mohamed Ben Abdellah University, Faculty of Science And Technology, Road Immouzer, BP 2202 Fez, Morocco; e Science and Technology of Lille USR 3290, Villeneuve d’ascq cedex, France; f Laboratory of Organic and Physical Chemistry, Applied Bioorganic Chemistry Team, Faculty of Sciences, Ibnou Zohr University, Agadir, Morocco; g Laboratory of Plant Chemistry, Organic and Bioorganic Synthesis, Faculty of Sciences, Mohammed V University in Rabat, 4 Avenue Ibn Battouta BP 1014 RP, Rabat, Morocco; Vienna University of Technology, Austria

**Keywords:** benzimidazol-2-one, crystal structure, triazole, C—H⋯π(ring) inter­action, hydrogen bond

## Abstract

The benzimidazole entity of the title mol­ecule, C_17_H_21_N_5_O, is almost planar (r.m.s. deviation = 0.0262 Å). In the crystal, bifurcated C—H⋯O hydrogen bonds link individual mol­ecules into layers extending parallel to the *ac* plane. Two weak C—H⋯π(ring) inter­actions may also be effective in the stabilization of the crystal structure. Hirshfeld surface analysis of the crystal structure reveals that the most important contributions for the crystal packing are from H⋯H (57.9%), H⋯C/C⋯H (18.1%) and H⋯O/O⋯H (14.9%) inter­actions. Hydrogen bonding and van der Waals inter­actions are the most dominant forces in the crystal packing. Evaluation of the electrostatic, dispersion and total energy frameworks indicate that the stabilization of the title compound is dominated *via* dispersion energy contributions. The mol­ecular structure optimized by density functional theory (DFT) at the B3LYP/6–311 G(d,p) level is compared with the experimentally determined mol­ecular structure in the solid state.

## Abstract 2

In the title mol­ecule, the benzimidazole entity is almost planar (r.m.s. deviation = 0.0262 Å), while the triazole ring is oriented almost perpendicular to the benzimidazole ring. In the crystal, bifurcated C—H⋯O hydrogen bonds link individual mol­ecules into layers extending parallel to the *ac* plane.

## 1. Chemical context

Heterocyclic compounds comprising the benzimidazolone fragment have attracted inter­est due to their remarkable usefulness in various therapeutic applications. Extensive research has revealed several pharmacological and biological properties associated with these compounds, including anti­proliferative (Guillon *et al.*, 2022 ▸), anti­bacterial (Al-Ghulikah *et al.*, 2023 ▸; Saber *et al.*, 2020 ▸), anti­cancer (Dimov *et al.*, 2021 ▸) and anti­viral (Ferro *et al.*, 2017 ▸) activities.

**Figure 1:**
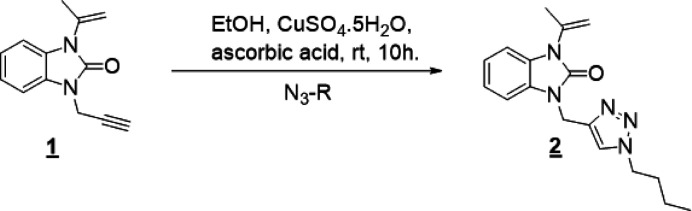
Schematic synthesis procedure for obtaining benzimidazol-2-one derivatives.

Our current studies focus on the syntheses of new benzimidazol-2-one derivatives by combining them with the 1,2,3-triazole moiety by using ‘click chemistry’. Specifically, the copper-catalysed azide-alkyne cyclo­addition (CuAAC) method has proved useful in obtaining the title compound, 1-[(1-butyl-1*H*-1,2,3-triazol-4-yl)meth­yl]-3-(prop-1-en-2-yl)-1*H*-benzimidazol-2-one (Fig. 1 ▸). In this context, we determined its crystal structure, performed a Hirshfeld surface analysis and calculated inter­molecular inter­action energies and energy frameworks. A comparison of the experimentally determined mol­ecular structure in the solid state with the mol­ecular structure optimized by using density functional theory (DFT) at the B3LYP/6-311G(d,p) level was also carried out. 
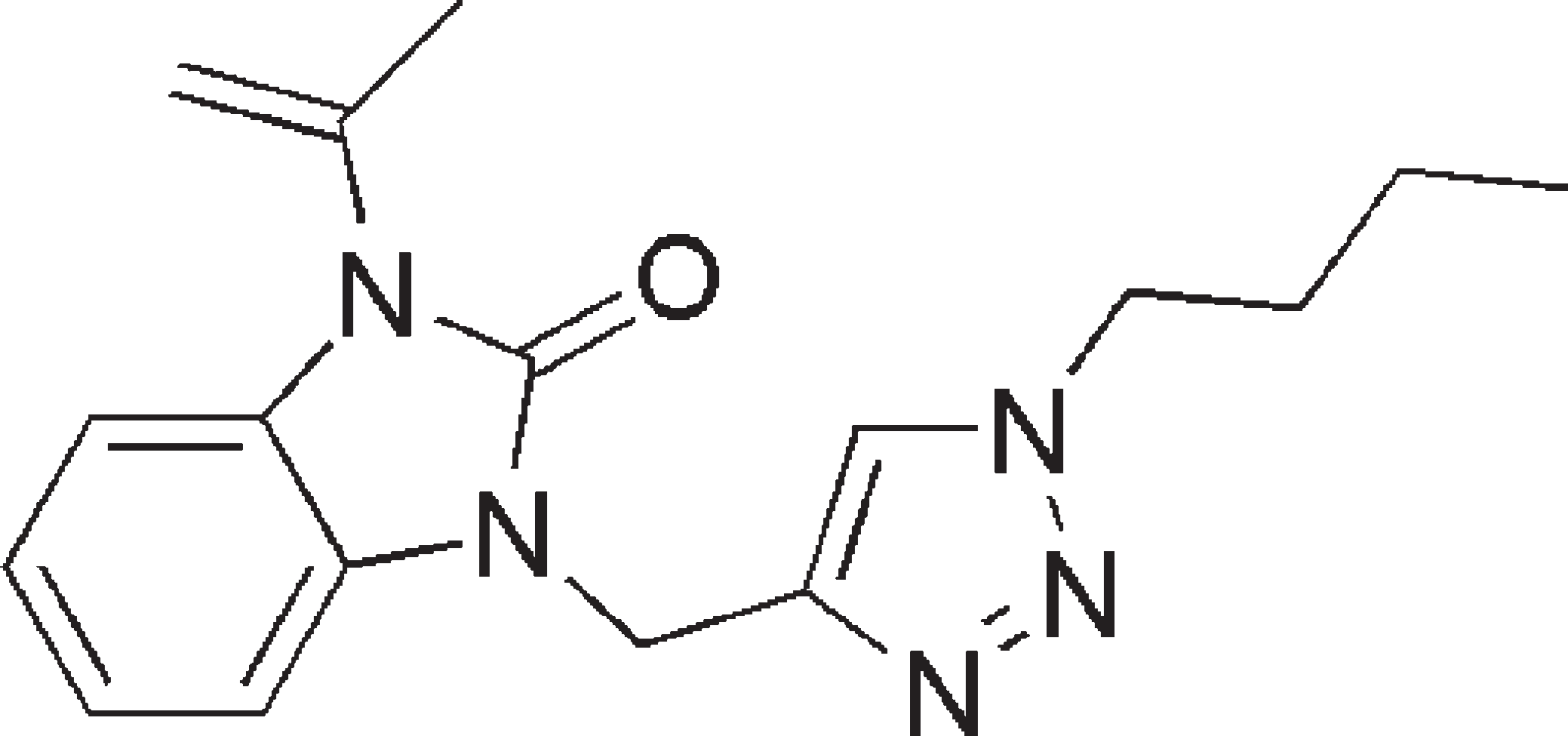


## 2. Structural commentary

**Figure 2:**
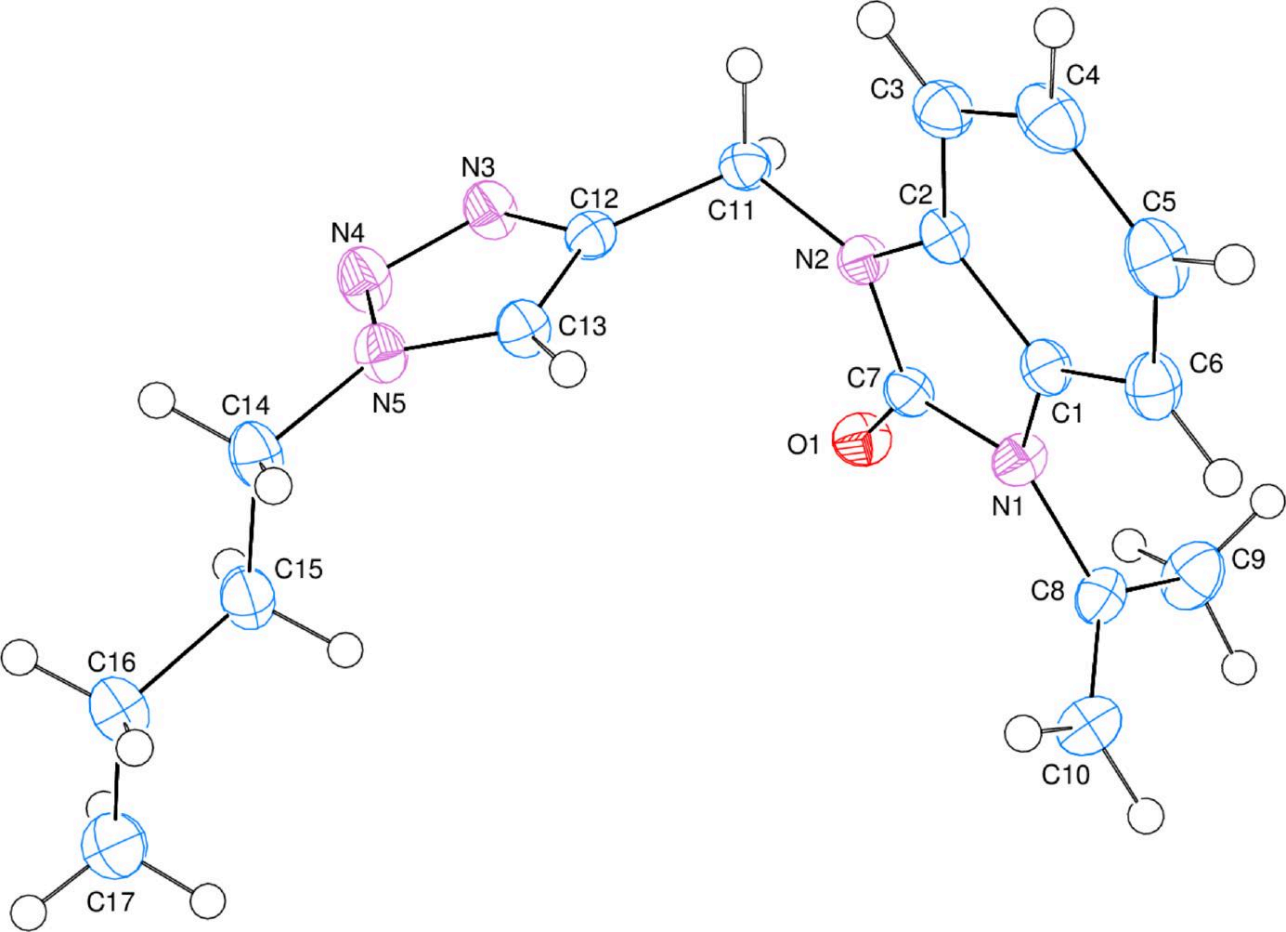
The title mol­ecule with the atom-labelling scheme and displacement ellipsoids drawn at the 50% probability level.

In the mol­ecular structure of the title compound (Fig. 2 ▸), the benzimidazole entity is almost planar (r.m.s. deviation of atoms C1–C7/N1–N2/O1 is 0.0262 Å); rings *A* (C1–C6) and *B* (N1/N2/C1/C2/C7) are oriented at a dihedral angle of 1.20 (4)°. The triazole ring *C* (N3–N5/C12/C13) is oriented almost perpendicular to the benzimidazole fragment with dihedral angles of *A*/*C* = 85.36 (4)° and *B*/*C* = 86.52 (4)°. Atoms O1, C8 and C11 are −0.0139 (9) Å, 0.0759 (12) Å and −0.0632 (12) Å away, while atoms C11 and C14 are 0.0245 (11) Å and −0.0835 (13) Å away from the best least-squares planes of rings *B* and *C*, respectively. Hence, they appear almost coplanar with the corresponding ring planes.

## 3. Supra­molecular features

**Figure 3:**
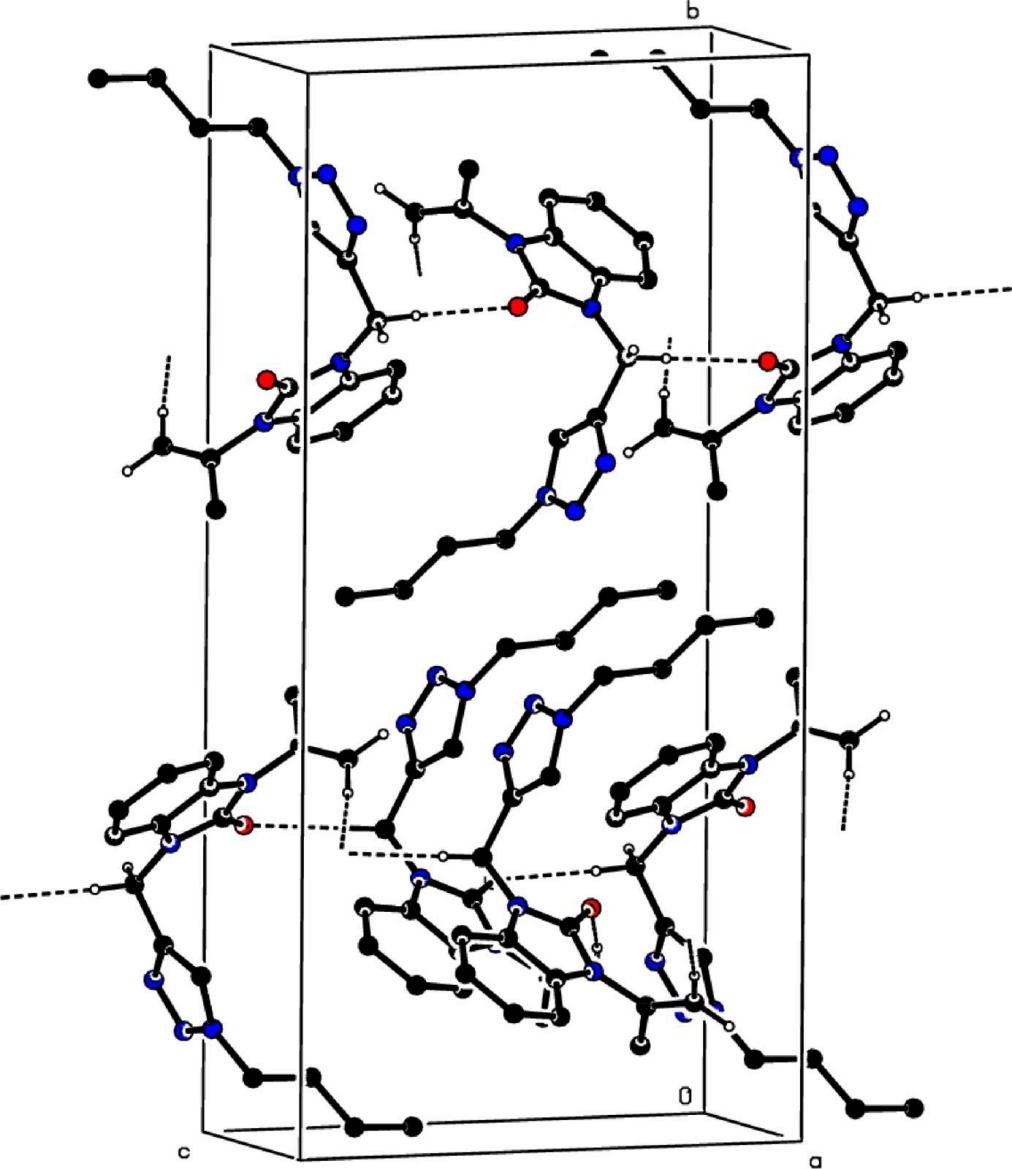
A partial packing diagram of the title compound viewed down the *a* axis. Non-inter­acting hydrogen atoms were omitted for clarity.

In the crystal, bifurcated C—H⋯O hydrogen bonds (Table 1 ▸, Fig. 3 ▸) link individual mol­ecules into layers extending parallel to the *ac* plane. Two weak C—H⋯π(ring) inter­actions (Table 1 ▸) may also be effective in the stabilization of the crystal packing.

## 4. Hirshfeld surface analysis

**Figure 4:**
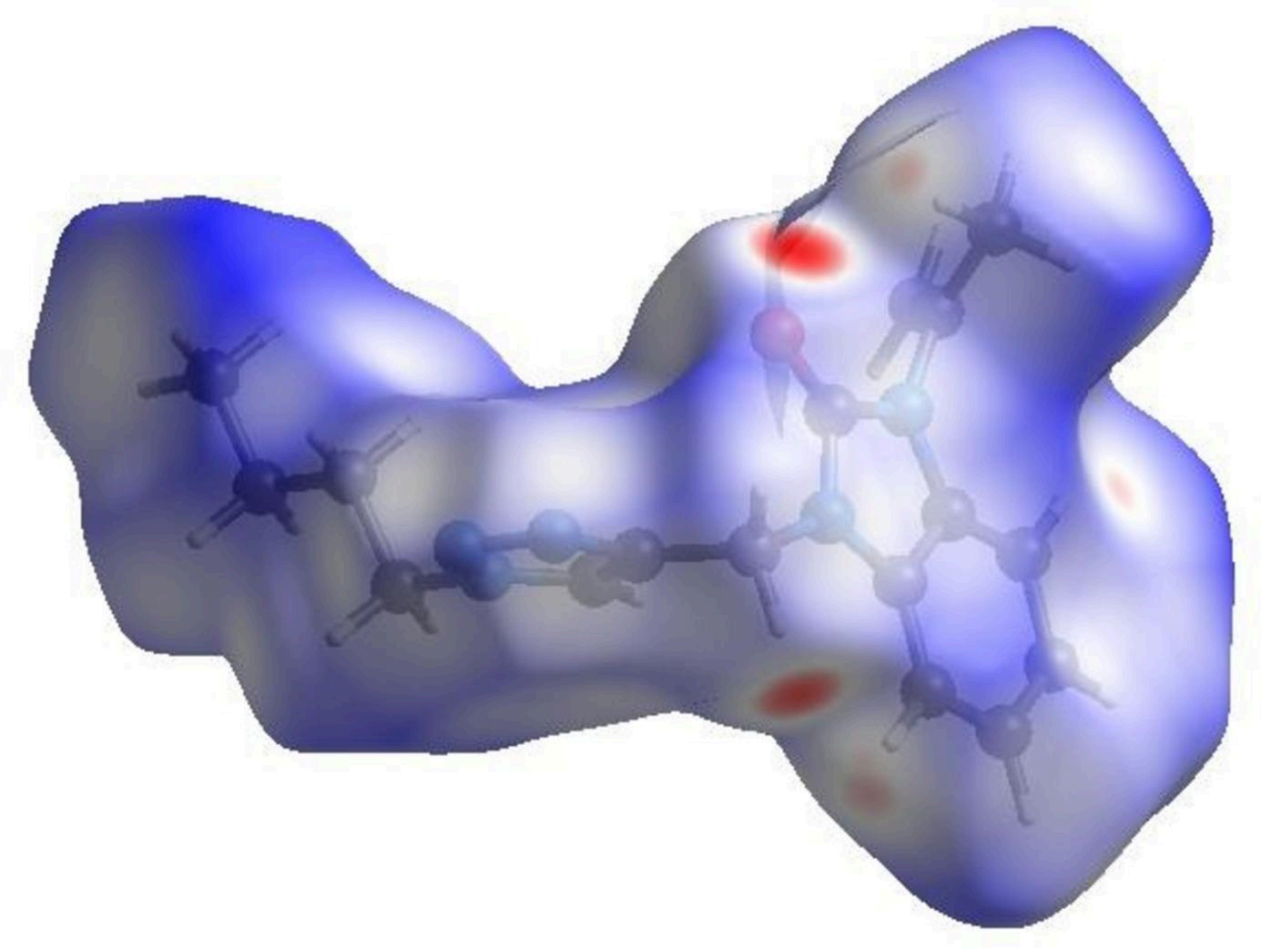
View of the three-dimensional HS of the title compound plotted over *d*_norm_.

**Figure 5:**
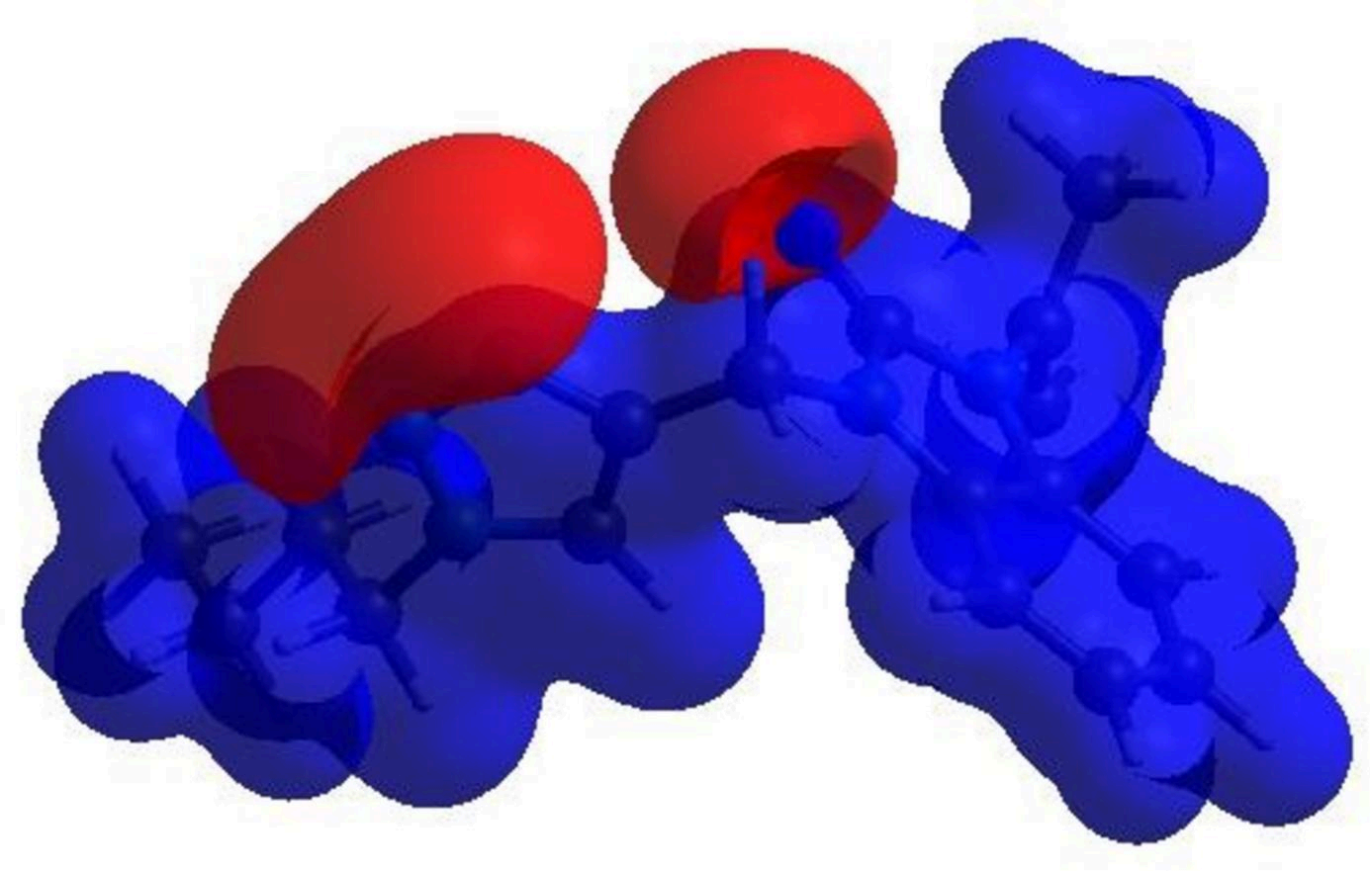
View of the three-dimensional HS of the title compound plotted over electrostatic potential energy using the STO-3 G basis set at the Hartree–Fock level of theory. Hydrogen-bond donors and acceptors are shown as blue and red regions around the atoms corresponding to positive and negative potentials, respectively.

**Figure 6:**
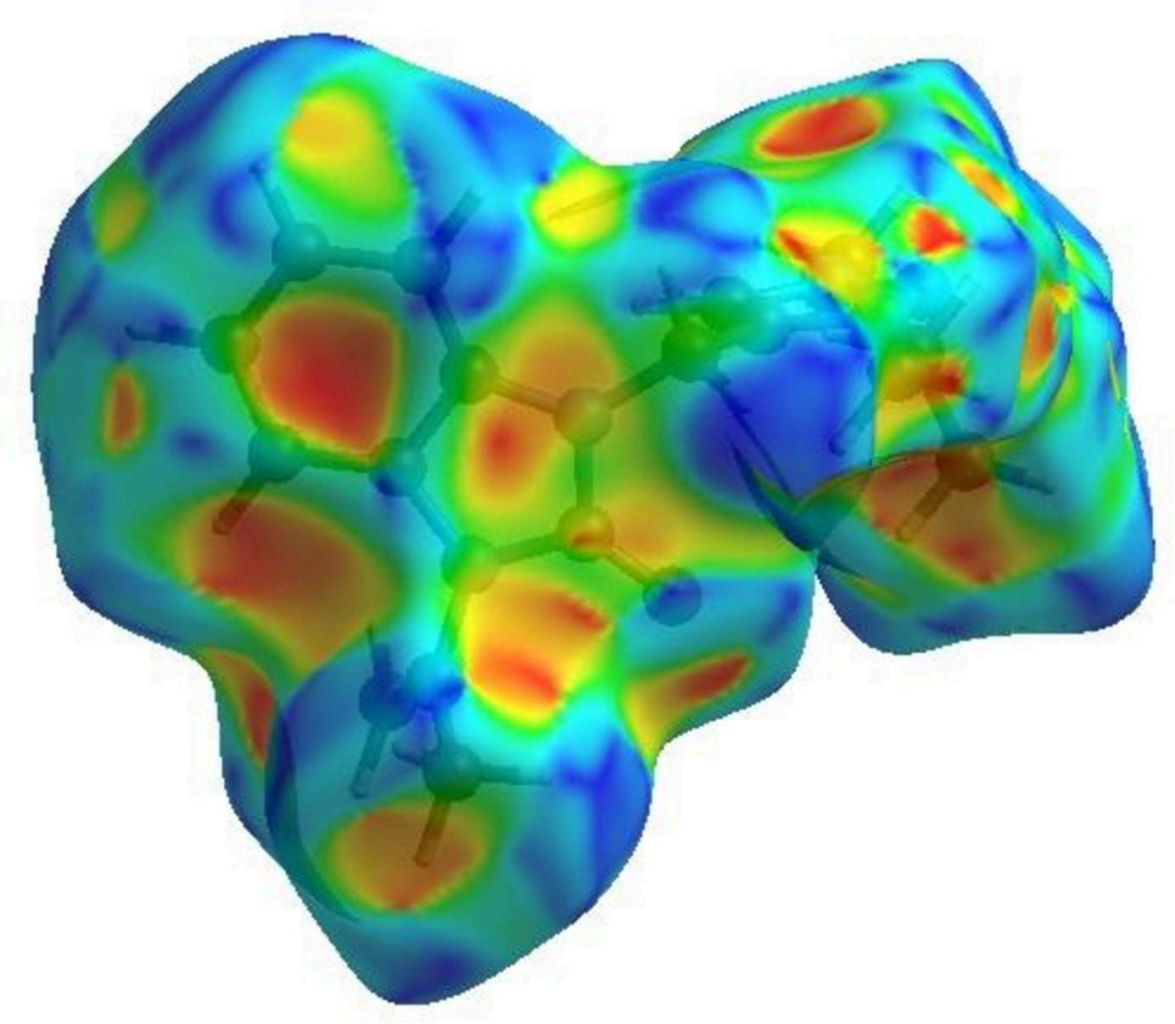
HS of the title compound plotted over shape-index.

**Figure 7:**
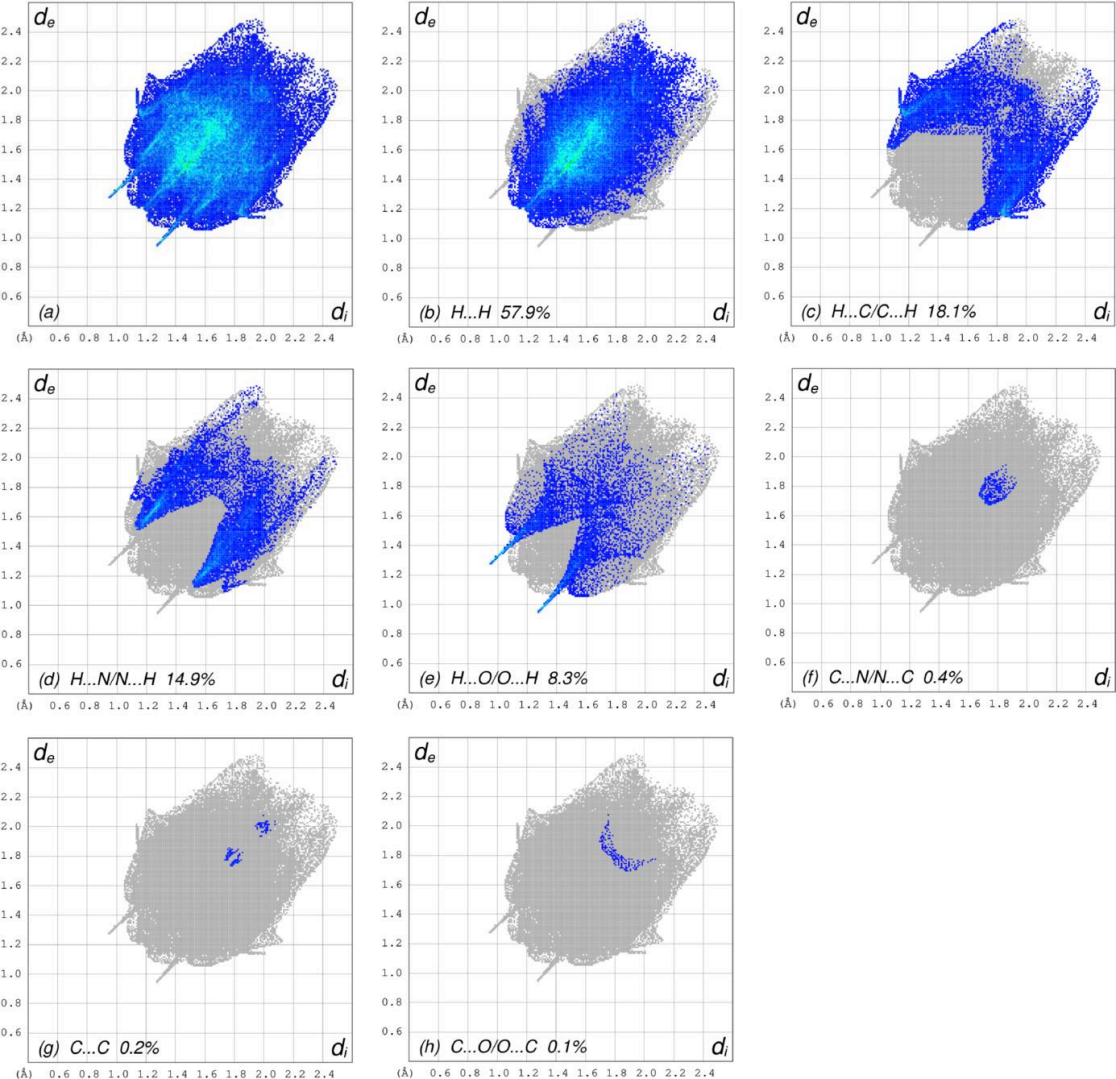
The full two-dimensional fingerprint plots for the title compound, showing (*a*) all inter­actions, and delineated into (*b*) H⋯H, (*c*) H⋯C/C⋯H, (*d*) H⋯N/N⋯H (*e*) H⋯O/O⋯H, (*f*) C⋯N/N⋯C, (*g*) C⋯C and (*h*) C⋯O/O⋯C inter­actions. The *d*_i_ and *d*_e_ values are the closest inter­nal and external distances (in Å) from given points on the Hirshfeld surface.

In order to visualize the inter­molecular inter­actions in the crystal structure of the title compound, a Hirshfeld surface (HS) analysis (Hirshfeld, 1977 ▸; Spackman & Jayatilaka, 2009 ▸) was carried out using *CrystalExplorer* (Spackman *et al.*, 2021 ▸). In the HS plotted over *d*_norm_ (Fig. 4 ▸), the white surface indicates contacts with distances equal to the sum of van der Waals radii, and the red and blue surfaces contacts shorter (in close contact) or longer (distinct contact), respectively, than the van der Waals radii (Venkatesan *et al.*, 2016 ▸). The bright-red spots indicate their roles as the respective donors and/or acceptors; they also appear as blue and red regions corresponding to positive and negative potentials on the HS mapped over electrostatic potential (Spackman *et al.*, 2008 ▸; Jayatilaka *et al.*, 2005 ▸), as shown in Fig. 5 ▸. The blue regions indicate positive electrostatic potential (hydrogen-bond donors), while the red regions indicate negative electrostatic potential (hydrogen-bond acceptors). The shape-index of the HS does not reveal any relevant π–π inter­actions (Fig. 6 ▸). However, the shape-index shows C—H⋯π inter­actions present as ‘red *p*-holes’, which are related to the electron ring inter­actions between the CH groups and the centroids of the aromatic rings of neighbouring mol­ecules (Table 1 ▸; Fig. 6 ▸). The overall two-dimensional fingerprint plot, Fig. 7 ▸ *a*, and those delineated into H⋯H, H⋯C/C⋯H, H⋯N/N⋯H, H⋯O/ O⋯H, C⋯N/N⋯C, C⋯C and C⋯O/O⋯C inter­actions (McKinnon *et al.*, 2007 ▸) are illustrated in Fig. 7 ▸ *b*–*h*, respectively, together with their relative contributions to the HS. The most important inter­action is H⋯H contributing with 57.9% to the overall crystal packing, which is reflected in Fig. 7 ▸ *b* as widely scattered points of high density due to the large hydrogen content of the mol­ecule with the tip at *d*_e_ = *d*_i_ = 1.20 Å. As a result of the presence of C—H⋯π inter­actions, the H⋯C/C⋯H contacts contribute 18.1% to the overall crystal packing, as reflected in Fig. 7 ▸ *c* with the tips at *d*_e_ + *d*_i_ = 2.66 Å. The symmetrical pair of wings in the fingerprint plot delineated into H⋯N/N⋯H contacts (Fig. 7 ▸ *d*), with 14.9% contribution to the HS, has the tips at *d*_e_ + *d*_i_ = 2.66 Å. The symmetrical pair of spikes in the fingerprint plot delineated into H⋯O/O⋯H contacts (Fig. 7 ▸ *e*), 8.3% contribution to the HS, have the tips at *d*_e_ + *d*_i_ = 2.22 Å. Finally, the C⋯N/N⋯C (Fig. 7 ▸ *f*), C⋯C (Fig. 7 ▸ *g*) and C⋯O/O⋯C (Fig. 7 ▸ *h*) inter­actions make small contibutions of 0.4%, 0.2% and 0.1%, respectively, to the HS.

**Figure 8:**
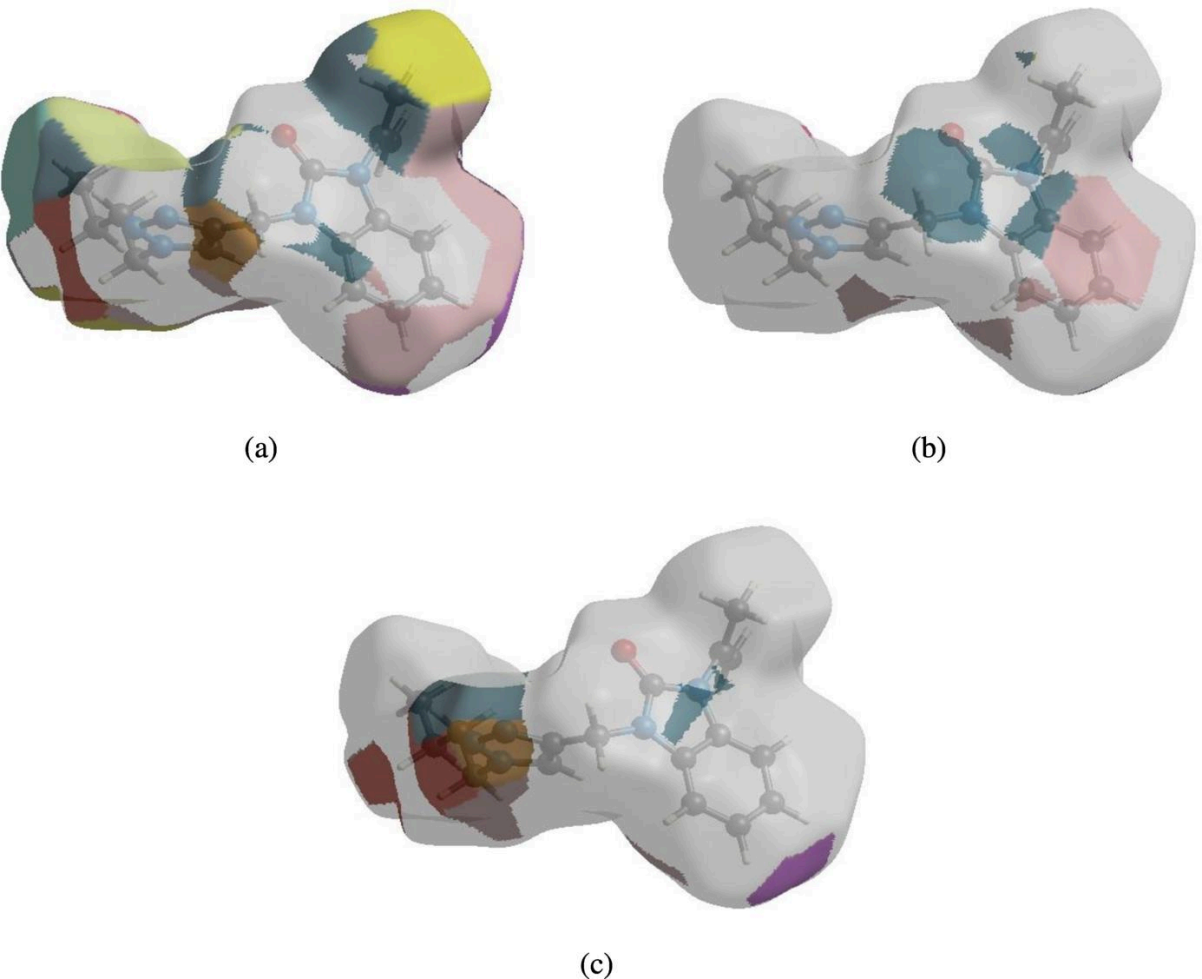
The HS representations with the function *d*_norm_ plotted onto the surface for (*a*) H⋯H, (*b*) H⋯C/C⋯H and (*c*) H⋯N/N⋯H inter­actions.

The nearest neighbour environment of a mol­ecule can be determined from the colour patches on the HS based on how close to other mol­ecules they are. The HS representations with the function *d*_norm_ plotted onto the surface are shown for the H⋯H, H⋯C/C⋯H and H⋯N/N⋯H inter­actions in Fig. 8 ▸ *a*–*c*, respectively. The HS analysis confirms the importance of H-atom contacts in establishing the packing. The large number of H⋯H, H⋯C/C⋯H and H⋯N/N⋯H inter­actions suggest that van der Waals and hydrogen-bonding inter­actions play the major roles in the crystal packing (Hathwar *et al.*, 2015 ▸).

## 5. Inter­action energy calculations and energy frameworks

**Figure 9:**
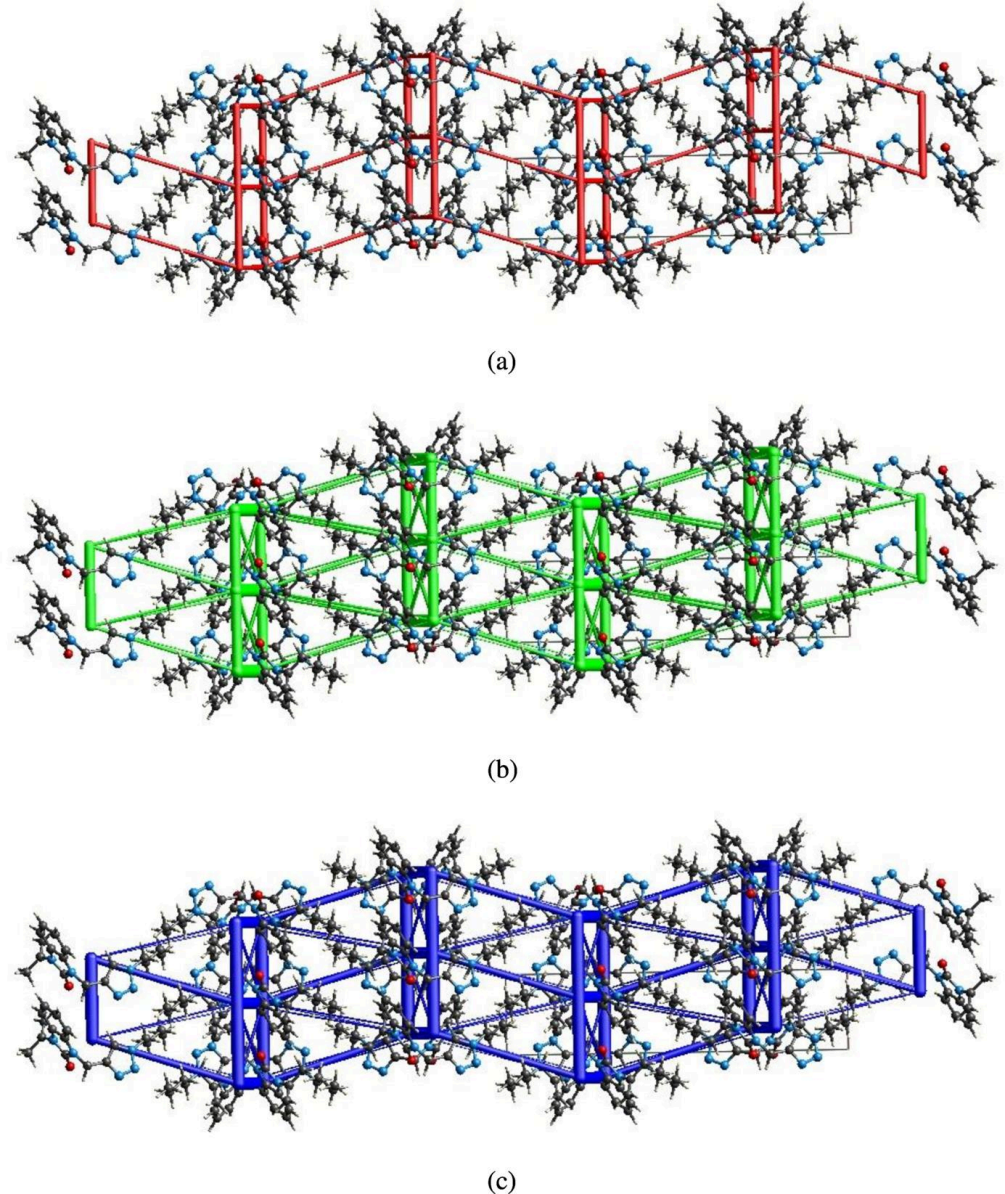
The energy frameworks for a cluster of mol­ecules of the title compound viewed down the *c* axis, showing (*a*) electrostatic energy, (*b*) dispersion energy and (*c*) total energy diagrams. The cylindrical radius is proportional to the relative strength of the corresponding energies and they were adjusted to the same scale factor of 80 with cut-off value of 5 kJ mol^−1^ within 2×2×2 unit cells.

The inter­molecular inter­action energies were calculated using the CE–B3LYP/6–311G(d,p) energy model available in *CrystalExplorer* (Spackman *et al.*, 2021 ▸), where a cluster of mol­ecules is generated by applying crystallographic symmetry operations with respect to a selected central mol­ecule within a radius of 3.8 Å by default (Turner *et al.*, 2014 ▸). The total inter­molecular energy (*E*_tot_) is the sum of electrostatic (*E*_ele_), polarization (*E*_pol_), dispersion (*E*_dis_) and exchange-repulsion (*E*_rep_) energies (Turner *et al.*, 2015 ▸) with scale factors of 1.057, 0.740, 0.871 and 0.618, respectively (Mackenzie *et al.*, 2017 ▸). Hydrogen-bonding inter­action energies (in kJ mol^−1^) were calculated as −32.1 (*E*_ele_), −9.4 (*E*_pol_), −53.7 (*E*_dis_), 48.4 (*E*_rep_) and −57.7 (*E*_tot_) for C10—H10*B*⋯O1 and −21.0 (*E*_ele_), −7.7 (*E*_pol_), −65.2 (*E*_dis_), 51.6 (E_rep_) and −52.8 (*E*_tot_) for the C11—H11*B*⋯O1 hydrogen bond. Energy frameworks combine the calculation of inter­molecular inter­action energies with a graphical representation of their magnitude (Turner *et al.*, 2015 ▸). Energies between mol­ecular pairs are represented as cylinders joining the centroids of pairs of mol­ecules with the cylinder radius proportional to the relative strength of the corresponding inter­action energy. Energy frameworks were constructed for *E*_ele_ (red cylinders), *E*_dis_ (green cylinders) and *E*_tot_ (blue cylinders) (Fig. 9 ▸ *a*–*c*). The evaluation of the electrostatic, dispersion and total energy frameworks indicate that the stabilization is dominated *via* dispersion energies in the crystal structure of the title compound.

## 6. DFT calculations

The mol­ecular structure in the gas phase was optimized using density functional theory (DFT) with the B3LYP functional and 6-311G(d,p) basis-set calculations, as implemented in GAUSSIAN 09 (Frisch *et al.* 2009 ▸). The optimized parameters, including bond lengths and angles, showed satisfactory agreement with the experimental structural data (Table 2 ▸). The largest differences between the calculated and experimental values were observed for the C1—N1 (0.04 Å), N1—C7 and N1—C8 (0.02 Å) bond lengths, the N4—N3—C12 (0.82°) bond angle and the torsion angle N3—C12—C13—N5 (0.3°). These differences may be due to the fact that the calculations are based on an isolated mol­ecule at 0 K, while the experimental results were obtained from inter­acting mol­ecules in the solid state, where intra- and inter­molecular inter­actions with neighbouring mol­ecules are present.

## 7. Database survey

**Figure 10:**
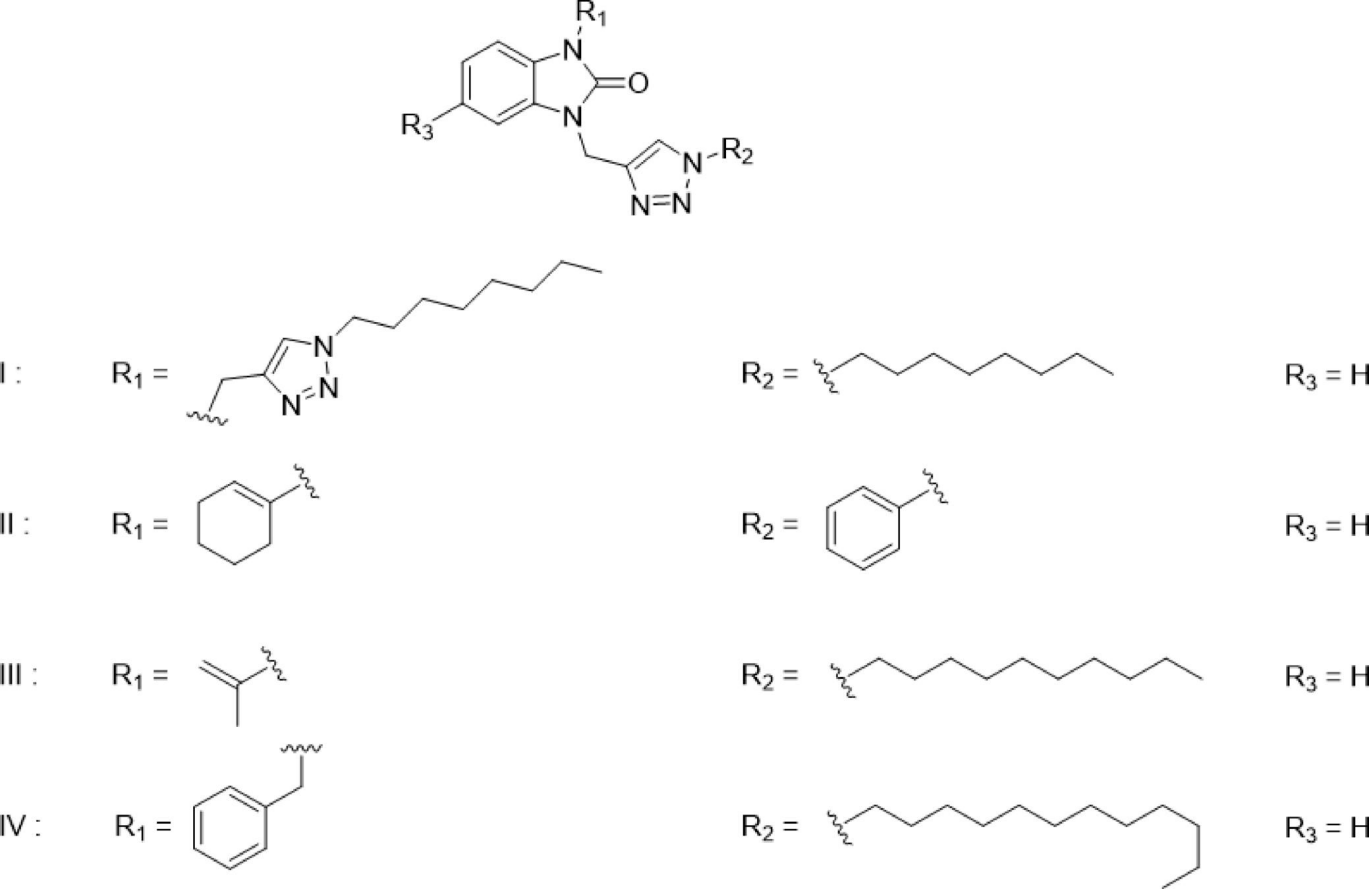
Related mol­ecular fragments for searching the CSD database.

A survey of the Cambridge Structural Database (CSD, updated March 2024; Groom *et al.*, 2016 ▸) indicates that there are several mol­ecules similar to the title compound (Fig. 10 ▸). These include I (CSD refcode YIVWUZ; Zouhair *et al.*, 2023 ▸), II with *R*_1_ = –C_6_H_9_, *R*_2_ = –C_6_H_5_ and *R*_3_ = H (CSD refcode PAZFOO; Adardour *et al.*, 2017 ▸), III with *R*_1_ = –C(CH_3_)=CH_2_, *R*_2_ = –C_10_H**_22_** and *R*_3_ = –H (CSD refcode ETAJOB; Saber *et al.*, 2021 ▸) and IV with *R*_1_ = –CH_2_C_6_H_5_, *R*_2_ = -C_12_H_26_ and *R*_3_ = H (CSD refcode ETAKAO; Saber *et al.*, 2021 ▸). The benzimidazol-2-one unit in all of these compounds is almost planar, with the dihedral angle between the constituent rings being less than 1°, or having the nitro­gen atom bearing the exocyclic substituent less than 0.03 Å from the mean plane of the remaining nine atoms.

## 8. Synthesis and crystallization

To a solution of 2.87 mmol of 1-(prop-1-en-2-yl)-3-(prop-2-yn­yl)-1*H*-benzimidazol-2-one and 0.45 mmol of 1-azido­butane in 10 ml of ethanol were added 1.64 mmol of CuSO_4_ and 3.73 mmol of sodium ascorbate dissolved in 10 ml of distilled water. The reaction mixture was stirred for 10 h at room temperature and monitored by TLC. After filtration and concentration of the solution under reduced pressure, the residue obtained was chromatographed on a silica gel column using ethyl acetate/hexane (3/1) as eluent. The resulting solid was filtered off, washed with water, dried, and then recrystallized from ethanol, yield: 73%.

## 9. Refinement

Crystal data, data collection and structure refinement details are summarized in Table 3 ▸. Methyl­ene hydrogens attached to C10 were located in a difference-Fourier map, and were included as riding contributions in idealized positions with *U*_iso_(H) = 1.2*U*_eq_(C). Aromatic H atoms were treated the same way, and methyl H atoms with *U*_iso_(H) = 1.5*U*_eq_(C).

## Supplementary Material

Crystal structure: contains datablock(s) I. DOI: 10.1107/S2056989024004043/wm5715sup1.cif

Structure factors: contains datablock(s) I. DOI: 10.1107/S2056989024004043/wm5715Isup2.hkl

Supporting information file. DOI: 10.1107/S2056989024004043/wm5715Isup3.cdx

Supporting information file. DOI: 10.1107/S2056989024004043/wm5715Isup4.cml

CCDC reference: 2352892

Additional supporting information:  crystallographic information; 3D view; checkCIF report

## Figures and Tables

**Table 1:** Hydrogen-bond geometry (Å, °) *Cg*1 is the centroid of the C1–C6 ring.

*D*—H⋯*A*	*D*—H	H⋯*A*	*D*⋯*A*	*D*—H⋯*A*
C10—H10*B*⋯O1^i^	0.987 (17)	2.303 (17)	3.2691 (16)	165.9 (13)
C11—H11*B*⋯O1^ii^	0.99	2.35	3.3346 (14)	171
C11—H11*A*⋯*Cg*1^i^	0.99	2.70	3.4833 (12)	136
C15—H15*A*⋯*Cg*1^iii^	0.99	2.90	3.8400 (13)	159

Symmetry codes: (i) 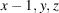 ; (ii) 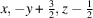 ; (iii) 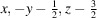 .

**Table 2:** Comparison of selected (X-ray and DFT) bond length and angles (Å, °)

Bonds/angles	X-ray	B3LYP/6–311G(d,p)
C1—N1	1.3992 (14)	1.352
N1—C7	1.3847 (14)	1.364
N1—C8	1.4402 (14)	1.461
O1—C7	1.2250 (13)	1.235
C2—N2	1.3919 (13)	1.378
N2—C7	1.3770 (14)	1.382
N2—C11	1.4568 (14)	1.438
N3—N4	1.3222 (14)	1.311
N4—N3—C12	108.94 (9)	108.12
N3—N4—N5	106.93 (9)	106.47
N4—N5—C13	111.07 (9)	111.64
N4—N5—C14	120.09 (9)	120.71
C13—N5—C14	128.71 (10)	128.48
N2—C7—N1	106.56 (9)	106.29
N3—N4—N5—C13	0.00 (13)	0.02
N3—C12—C13—N5	–0.12 (12)	0.18
C1—N1—C7—O1	–178.68 (11)	–178.74
C1—N1—C7—N2	0.08 (12)	0.07

**Table 3:** Experimental details

Crystal data	
Chemical formula	C_17_H_21_N_5_O
*M*_r_	311.39
Crystal system, space group	Monoclinic, *P*2_1_/*c*
Temperature (K)	160
*a*, *b*, *c* (Å)	5.7032 (1), 24.2184 (5), 11.8683 (2)
β (°)	91.312 (2)
*V* (Å^3^)	1638.85 (5)
*Z*	4
Radiation type	Cu *K*α
μ (mm^−1^)	0.66
Crystal size (mm)	0.19 × 0.12 × 0.05

Data collection	
Diffractometer	SuperNova, Dual, Cu at home/near, Atlas
Absorption correction	Analytical [*CrysAlis PRO* (Rigaku OD, 2023 ▸) using a multifaceted crystal model (Clark & Reid, 1995 ▸)]
*T*_min_, *T*_max_	0.894, 0.971
No. of measured, independent and observed [*I* > 2σ(*I*)] reflections	19825, 3435, 2968
*R*_int_	0.034
(sin θ/λ)_max_ (Å^−1^)	0.631

Refinement	
*R*[*F* ^2^ > 2σ(*F* ^2^)], *wR*(*F* ^2^), *S*	0.034, 0.086, 1.05
No. of reflections	3435
No. of parameters	219
H-atom treatment	H atoms treated by a mixture of independent and constrained refinement
Δρ_max_, Δρ_min_ (e Å^−3^)	0.22, −0.21

Computer programs: *CrysAlis PRO* (Rigaku OD, 2023 ▸), *SHELXT* (Sheldrick, 2015*a* ▸), *SHELXL* (Sheldrick, 2015*b* ▸), *OLEX2* (Dolomanov *et al.*, 2009 ▸) and *publCIF* (Westrip, 2010 ▸).

## supplementary crystallographic information

### Crystal data

**Table:**

C_17_H_21_N_5_O	*F*(000) = 664
*M**_r_* = 311.39	*D*_x_ = 1.262 Mg m^−^^3^
Monoclinic, *P*2_1_/*c*	Cu *K*α radiation, λ = 1.54184 Å
*a* = 5.7032 (1) Å	Cell parameters from 10273 reflections
*b* = 24.2184 (5) Å	θ = 3.6–76.4°
*c* = 11.8683 (2) Å	µ = 0.66 mm^−^^1^
β = 91.312 (2)°	*T* = 160 K
*V* = 1638.85 (5) Å^3^	Plate, colourless
*Z* = 4	0.19 × 0.12 × 0.05 mm

### Data collection

**Table:**

SuperNova, Dual, Cu at home/near, Atlas diffractometer	3435 independent reflections
Radiation source: micro-focus sealed X-ray tube, SuperNova (Cu) X-ray Source	2968 reflections with *I* > 2σ(*I*)
Mirror monochromator	*R*_int_ = 0.034
Detector resolution: 10.3801 pixels mm^-1^	θ_max_ = 76.8°, θ_min_ = 3.7°
ω scans	*h* = −7→7
Absorption correction: analytical [CrysAlisPro (Rigaku OD, 2023) using a multifaceted crystal model (Clark & Reid, 1995)]	*k* = −30→26
*T*_min_ = 0.894, *T*_max_ = 0.971	*l* = −14→14
19825 measured reflections	

### Refinement

**Table:**

Refinement on *F*^2^	Hydrogen site location: mixed
Least-squares matrix: full	H atoms treated by a mixture of independent and constrained refinement
*R*[*F*^2^ > 2σ(*F*^2^)] = 0.034	*w* = 1/[σ^2^(*F*_o_^2^) + (0.0353*P*)^2^ + 0.4935*P*] where *P* = (*F*_o_^2^ + 2*F*_c_^2^)/3
*wR*(*F*^2^) = 0.086	(Δ/σ)_max_ = 0.001
*S* = 1.05	Δρ_max_ = 0.22 e Å^−^^3^
3435 reflections	Δρ_min_ = −0.21 e Å^−^^3^
219 parameters	Extinction correction: SHELXL (Sheldrick, 2015b), Fc^*^=kFc[1+0.001xFc^2^λ^3^/sin(2θ)]^-1/4^
0 restraints	Extinction coefficient: 0.0018 (2)
Primary atom site location: dual	

### Special details

**Table:**

Geometry. All esds (except the esd in the dihedral angle between two l.s. planes) are estimated using the full covariance matrix. The cell esds are taken into account individually in the estimation of esds in distances, angles and torsion angles; correlations between esds in cell parameters are only used when they are defined by crystal symmetry. An approximate (isotropic) treatment of cell esds is used for estimating esds involving l.s. planes.

### Fractional atomic coordinates and isotropic or equivalent isotropic displacement parameters (Å^2^)

**Table:**

	*x*	*y*	*z*	*U*_iso_*/*U*_eq_	
C1	0.57633 (19)	0.82949 (4)	0.42568 (9)	0.0208 (2)	
N1	0.71001 (16)	0.82797 (4)	0.52597 (8)	0.0223 (2)	
O1	1.04428 (14)	0.77946 (4)	0.58607 (6)	0.02597 (19)	
C2	0.68397 (19)	0.79364 (4)	0.35005 (9)	0.0202 (2)	
N2	0.87645 (16)	0.77053 (4)	0.40675 (7)	0.0203 (2)	
N3	1.17211 (17)	0.63652 (4)	0.43348 (9)	0.0262 (2)	
C3	0.5972 (2)	0.78704 (5)	0.24096 (9)	0.0252 (2)	
H3	0.671876	0.763257	0.189186	0.030*	
C4	0.3964 (2)	0.81658 (5)	0.21003 (10)	0.0298 (3)	
H4	0.332148	0.812748	0.135891	0.036*	
N4	1.08726 (18)	0.59076 (4)	0.47758 (9)	0.0300 (2)	
C5	0.2882 (2)	0.85168 (5)	0.28603 (11)	0.0302 (3)	
H5	0.151175	0.871223	0.262565	0.036*	
N5	0.85618 (17)	0.59867 (4)	0.48951 (8)	0.0245 (2)	
C6	0.3761 (2)	0.85880 (5)	0.39558 (10)	0.0264 (2)	
H6	0.301892	0.882696	0.447298	0.032*	
C7	0.89510 (19)	0.79157 (5)	0.51438 (9)	0.0206 (2)	
H10A	0.459 (3)	0.8759 (7)	0.7493 (13)	0.037 (4)*	
H10B	0.365 (3)	0.8256 (7)	0.6610 (13)	0.041 (4)*	
C8	0.6746 (2)	0.86076 (5)	0.62539 (9)	0.0235 (2)	
C9	0.8628 (2)	0.90232 (6)	0.65046 (12)	0.0349 (3)	
H9A	1.013615	0.883371	0.660827	0.052*	
H9B	0.825804	0.922450	0.719442	0.052*	
H9C	0.872346	0.928362	0.587528	0.052*	
C10	0.4838 (2)	0.85325 (5)	0.68373 (11)	0.0296 (3)	
C11	1.03801 (19)	0.72874 (5)	0.36645 (9)	0.0221 (2)	
H11A	1.200895	0.740317	0.384875	0.027*	
H11B	1.021480	0.725930	0.283427	0.027*	
C12	0.99452 (19)	0.67315 (5)	0.41755 (9)	0.0201 (2)	
C13	0.79133 (19)	0.64923 (5)	0.45341 (9)	0.0230 (2)	
H13	0.638578	0.664893	0.452876	0.028*	
C14	0.7134 (2)	0.55616 (5)	0.54332 (11)	0.0289 (3)	
H14A	0.746715	0.519827	0.508967	0.035*	
H14B	0.545234	0.564507	0.529705	0.035*	
C15	0.7638 (2)	0.55325 (5)	0.66936 (11)	0.0297 (3)	
H15A	0.733683	0.589859	0.703238	0.036*	
H15B	0.931469	0.544300	0.682716	0.036*	
C16	0.6139 (3)	0.51012 (6)	0.72697 (12)	0.0392 (3)	
H16A	0.446195	0.519093	0.713803	0.047*	
H16B	0.643818	0.473503	0.693086	0.047*	
C17	0.6652 (3)	0.50730 (6)	0.85305 (13)	0.0462 (4)	
H17A	0.832201	0.499571	0.866492	0.069*	
H17B	0.571337	0.477838	0.886196	0.069*	
H17C	0.625225	0.542686	0.887757	0.069*	

### Atomic displacement parameters (Å^2^)

**Table:**

	*U*^11^	*U*^22^	*U*^33^	*U*^12^	*U*^13^	*U*^23^
C1	0.0202 (5)	0.0196 (5)	0.0226 (5)	−0.0020 (4)	−0.0017 (4)	0.0017 (4)
N1	0.0207 (4)	0.0248 (5)	0.0214 (5)	0.0030 (4)	−0.0015 (3)	−0.0022 (4)
O1	0.0235 (4)	0.0322 (4)	0.0221 (4)	0.0034 (3)	−0.0036 (3)	0.0013 (3)
C2	0.0193 (5)	0.0188 (5)	0.0223 (5)	−0.0020 (4)	−0.0008 (4)	0.0037 (4)
N2	0.0203 (4)	0.0214 (5)	0.0193 (4)	0.0022 (4)	−0.0003 (3)	0.0006 (3)
N3	0.0205 (5)	0.0255 (5)	0.0329 (5)	0.0027 (4)	0.0022 (4)	0.0050 (4)
C3	0.0283 (6)	0.0252 (6)	0.0220 (5)	−0.0036 (5)	−0.0017 (4)	0.0010 (4)
C4	0.0309 (6)	0.0318 (6)	0.0262 (6)	−0.0047 (5)	−0.0081 (5)	0.0067 (5)
N4	0.0223 (5)	0.0254 (5)	0.0424 (6)	0.0038 (4)	0.0038 (4)	0.0068 (4)
C5	0.0233 (6)	0.0291 (6)	0.0379 (7)	0.0002 (5)	−0.0072 (5)	0.0086 (5)
N5	0.0203 (5)	0.0228 (5)	0.0302 (5)	−0.0011 (4)	0.0002 (4)	0.0026 (4)
C6	0.0224 (5)	0.0235 (6)	0.0332 (6)	0.0016 (4)	−0.0008 (4)	0.0028 (5)
C7	0.0195 (5)	0.0215 (5)	0.0210 (5)	−0.0009 (4)	0.0010 (4)	0.0019 (4)
C8	0.0248 (5)	0.0214 (5)	0.0241 (5)	0.0025 (4)	−0.0027 (4)	−0.0035 (4)
C9	0.0308 (6)	0.0317 (7)	0.0422 (7)	−0.0055 (5)	−0.0005 (5)	−0.0098 (5)
C10	0.0275 (6)	0.0325 (6)	0.0290 (6)	0.0029 (5)	0.0018 (5)	−0.0057 (5)
C11	0.0221 (5)	0.0224 (5)	0.0219 (5)	0.0022 (4)	0.0042 (4)	0.0016 (4)
C12	0.0198 (5)	0.0220 (5)	0.0186 (5)	0.0017 (4)	−0.0012 (4)	−0.0008 (4)
C13	0.0189 (5)	0.0234 (5)	0.0268 (5)	0.0019 (4)	−0.0007 (4)	0.0022 (4)
C14	0.0259 (6)	0.0237 (6)	0.0372 (7)	−0.0056 (5)	0.0001 (5)	0.0038 (5)
C15	0.0291 (6)	0.0242 (6)	0.0358 (7)	−0.0014 (5)	0.0025 (5)	0.0029 (5)
C16	0.0488 (8)	0.0292 (7)	0.0401 (7)	−0.0074 (6)	0.0097 (6)	0.0037 (5)
C17	0.0656 (10)	0.0335 (7)	0.0399 (8)	0.0074 (7)	0.0122 (7)	0.0062 (6)

### Geometric parameters (Å, º)

**Table:**

C1—N1	1.3992 (14)	C8—C10	1.3158 (17)
C1—C2	1.4007 (15)	C9—H9A	0.9800
C1—C6	1.3847 (16)	C9—H9B	0.9800
N1—C7	1.3847 (14)	C9—H9C	0.9800
N1—C8	1.4402 (14)	C10—H10A	0.966 (16)
O1—C7	1.2250 (13)	C10—H10B	0.987 (17)
C2—N2	1.3919 (13)	C11—H11A	0.9900
C2—C3	1.3846 (16)	C11—H11B	0.9900
N2—C7	1.3770 (14)	C11—C12	1.4995 (15)
N2—C11	1.4568 (14)	C12—C13	1.3719 (15)
N3—N4	1.3222 (14)	C13—H13	0.9500
N3—C12	1.3564 (14)	C14—H14A	0.9900
C3—H3	0.9500	C14—H14B	0.9900
C3—C4	1.3924 (17)	C14—C15	1.5184 (17)
C4—H4	0.9500	C15—H15A	0.9900
C4—C5	1.3938 (19)	C15—H15B	0.9900
N4—N5	1.3424 (13)	C15—C16	1.5218 (17)
C5—H5	0.9500	C16—H16A	0.9900
C5—C6	1.3933 (17)	C16—H16B	0.9900
N5—C13	1.3464 (15)	C16—C17	1.520 (2)
N5—C14	1.4678 (15)	C17—H17A	0.9800
C6—H6	0.9500	C17—H17B	0.9800
C8—C9	1.4965 (16)	C17—H17C	0.9800
			
N1—C1—C2	106.90 (9)	H9B—C9—H9C	109.5
C6—C1—N1	131.54 (11)	H10A—C10—H10B	119.5 (13)
C6—C1—C2	121.57 (10)	C8—C10—H10A	119.0 (9)
C1—N1—C8	126.77 (9)	C8—C10—H10B	121.5 (9)
C7—N1—C1	109.51 (9)	N2—C11—H11A	109.1
C7—N1—C8	123.64 (9)	N2—C11—H11B	109.1
N2—C2—C1	106.96 (9)	N2—C11—C12	112.28 (9)
C3—C2—C1	121.22 (10)	H11A—C11—H11B	107.9
C3—C2—N2	131.81 (11)	C12—C11—H11A	109.1
C2—N2—C11	128.19 (9)	C12—C11—H11B	109.1
C7—N2—C2	110.06 (9)	N3—C12—C11	120.90 (10)
C7—N2—C11	121.71 (9)	N3—C12—C13	108.30 (10)
N4—N3—C12	108.94 (9)	C13—C12—C11	130.79 (10)
C2—C3—H3	121.3	N5—C13—C12	104.77 (10)
C2—C3—C4	117.47 (11)	N5—C13—H13	127.6
C4—C3—H3	121.3	C12—C13—H13	127.6
C3—C4—H4	119.5	N5—C14—H14A	109.3
C3—C4—C5	121.10 (11)	N5—C14—H14B	109.3
C5—C4—H4	119.5	N5—C14—C15	111.53 (10)
N3—N4—N5	106.93 (9)	H14A—C14—H14B	108.0
C4—C5—H5	119.2	C15—C14—H14A	109.3
C6—C5—C4	121.62 (11)	C15—C14—H14B	109.3
C6—C5—H5	119.2	C14—C15—H15A	109.1
N4—N5—C13	111.07 (9)	C14—C15—H15B	109.1
N4—N5—C14	120.09 (9)	C14—C15—C16	112.28 (11)
C13—N5—C14	128.71 (10)	H15A—C15—H15B	107.9
C1—C6—C5	117.01 (11)	C16—C15—H15A	109.1
C1—C6—H6	121.5	C16—C15—H15B	109.1
C5—C6—H6	121.5	C15—C16—H16A	109.2
O1—C7—N1	127.03 (10)	C15—C16—H16B	109.2
O1—C7—N2	126.40 (10)	H16A—C16—H16B	107.9
N2—C7—N1	106.56 (9)	C17—C16—C15	112.07 (12)
N1—C8—C9	114.85 (10)	C17—C16—H16A	109.2
C10—C8—N1	119.21 (11)	C17—C16—H16B	109.2
C10—C8—C9	125.91 (11)	C16—C17—H17A	109.5
C8—C9—H9A	109.5	C16—C17—H17B	109.5
C8—C9—H9B	109.5	C16—C17—H17C	109.5
C8—C9—H9C	109.5	H17A—C17—H17B	109.5
H9A—C9—H9B	109.5	H17A—C17—H17C	109.5
H9A—C9—H9C	109.5	H17B—C17—H17C	109.5
			
C1—N1—C7—O1	−178.68 (11)	C3—C2—N2—C11	−4.13 (19)
C1—N1—C7—N2	0.08 (12)	C3—C4—C5—C6	0.16 (19)
C1—N1—C8—C9	−112.17 (13)	C4—C5—C6—C1	0.11 (18)
C1—N1—C8—C10	66.21 (16)	N4—N3—C12—C11	−178.92 (10)
C1—C2—N2—C7	−1.17 (12)	N4—N3—C12—C13	0.13 (13)
C1—C2—N2—C11	176.71 (10)	N4—N5—C13—C12	0.08 (13)
C1—C2—C3—C4	−1.20 (17)	N4—N5—C14—C15	71.86 (14)
N1—C1—C2—N2	1.18 (12)	N5—C14—C15—C16	178.92 (11)
N1—C1—C2—C3	−178.09 (10)	C6—C1—N1—C7	179.65 (12)
N1—C1—C6—C5	178.57 (12)	C6—C1—N1—C8	−3.6 (2)
C2—C1—N1—C7	−0.79 (12)	C6—C1—C2—N2	−179.20 (10)
C2—C1—N1—C8	175.93 (10)	C6—C1—C2—C3	1.53 (17)
C2—C1—C6—C5	−0.93 (17)	C7—N1—C8—C9	64.12 (15)
C2—N2—C7—N1	0.68 (12)	C7—N1—C8—C10	−117.50 (13)
C2—N2—C7—O1	179.46 (11)	C7—N2—C11—C12	72.83 (13)
C2—N2—C11—C12	−104.83 (12)	C8—N1—C7—O1	4.47 (18)
C2—C3—C4—C5	0.38 (18)	C8—N1—C7—N2	−176.77 (10)
N2—C2—C3—C4	179.74 (11)	C11—N2—C7—N1	−177.36 (9)
N2—C11—C12—N3	−150.31 (10)	C11—N2—C7—O1	1.41 (17)
N2—C11—C12—C13	30.88 (16)	C11—C12—C13—N5	178.80 (11)
N3—N4—N5—C13	0.00 (13)	C12—N3—N4—N5	−0.08 (13)
N3—N4—N5—C14	−176.20 (10)	C13—N5—C14—C15	−103.59 (14)
N3—C12—C13—N5	−0.12 (12)	C14—N5—C13—C12	175.86 (11)
C3—C2—N2—C7	177.99 (12)	C14—C15—C16—C17	179.91 (12)

### Hydrogen-bond geometry (Å, º)

*Cg*1 is the centroid of the C1–C6 ring.

**Table:**

*D*—H···*A*	*D*—H	H···*A*	*D*···*A*	*D*—H···*A*
C10—H10*B*···O1^i^	0.987 (17)	2.303 (17)	3.2691 (16)	165.9 (13)
C11—H11*B*···O1^ii^	0.99	2.35	3.3346 (14)	171
C11—H11*A*···*Cg*1^i^	0.99	2.70	3.4833 (12)	136
C15—H15*A*···*Cg*1^iii^	0.99	2.90	3.8400 (13)	159

Symmetry codes: (i) *x*−1, *y*, *z*; (ii) *x*, −*y*+3/2, *z*−1/2; (iii) *x*, −*y*−1/2, *z*−3/2.
